# Parameter-free dual-student ensemble for semi-supervised ischemic stroke segmentation on follow-up NCCT

**DOI:** 10.3389/fneur.2026.1910269

**Published:** 2026-09-22

**Authors:** Yuqing Sha, Long Wang, Chao Huang, Ximing Nie

**Affiliations:** 1 School of Computer and Communication Engineering, University of Science and Technology Beijing, Beijing, China; 2 Department of Neurology, Beijing Tiantan Hospital, Capital Medical University, Beijing, China

**Keywords:** deep learning, ensemble learning, ischemic stroke, non-contrast computed tomography, semi-supervised segmentation

## Abstract

**Introduction:**

Non-contrast computed tomography (NCCT) is widely used for post-treatment monitoring of patients with ischemic stroke due to its rapid acquisition, low cost, and non-invasiveness. Segmentation of ischemic lesions on NCCT images is critical for assessing disease severity and guiding clinical decision-making.

**Methods:**

We propose an ensemble-based semi-supervised segmentation (ESS) framework to reduce the dependence of convolutional neural networks (CNNs) on a large number of manual annotations. The proposed method mainly consists of two components: first, strong data augmentation is introduced to increase sample diversity and alleviate overfitting in the presence of limited labeled data; second, a parameter-free ensemble module is adopted to generate more stable supervision signals for unlabeled samples and improve the reliability of pseudo-labels by fusing the predictions of the two student models. The proposed ESS is evaluated on a manually annotated follow-up NCCT dataset consisting of 9,020 image slices.

**Results:**

ESS consistently achieves the highest Dice coefficient among all compared methods under all four labeled-data ratios, with a more pronounced performance advantage when labeled data are scarce.

**Discussion:**

These results demonstrate the effectiveness of ESS for semi-supervised segmentation of follow-up NCCT under limited annotation conditions.

## 1 Introduction

Stroke is one of the leading causes of death and long-term disability worldwide, imposing a substantial burden on public health systems (1, 2). Strokes can be classified into two categories: hemorrhagic and ischemic. Ischemic stroke refers to the sudden interruption of blood supply to the brain, accounting for approximately 80–90% of all stroke cases (3). Accurately identifying the location and volume of ischemic lesions is crucial for clinical decision-making and prognosis. Non-contrast computed tomography (NCCT) is widely used for imaging evaluation in stroke centers owing to its low cost, high efficiency, wide accessibility and non-invasiveness (4, 5). However, NCCT has limited image resolution, and early ischemic changes are often subtle and difficult to detect, such as loss of gray-white matter differentiation and disappearance of the insular ribbon (6, 7), making reliable interpretation highly dependent on experienced neuroradiologists (6). In developing countries and resource-limited regions, the shortage of specialized neuroradiology personnel is particularly pronounced (8). Therefore, developing an accurate, robust, and fully automatic method for NCCT-based ischemic lesion segmentation holds significant clinical importance.

In recent years, medical image segmentation methods based on convolutional neural networks (CNNs) have achieved remarkable progress (9). For stroke imaging, researchers have proposed various CNN models for CT perfusion (CTP), magnetic resonance imaging (MRI) and non-contrast CT (NCCT) (10–15). Recent studies have further explored specialized network architectures for ischemic stroke lesion segmentation. For example, 3D CoAt U SegNet (16) adopts a 3D deep learning architecture for acute ischemic stroke lesion segmentation on NCCT, while DB-Net (17) employs a dual-branch hybrid architecture combining CNN and Transformer representations for acute ischemic stroke lesion segmentation on NCCT. An automated deep U-Net model (18) has also been developed for sub-acute ischemic stroke lesion segmentation on FLAIR MRI. These studies have further advanced supervised stroke lesion segmentation. However, existing studies have mainly focused on acute or sub-acute stroke imaging, whereas relatively few investigations have addressed ischemic lesion segmentation on follow-up NCCT. Compared with the acute phase, lesions on follow-up NCCT may present changes such as edema resolution and encephalomalacia formation, and may exhibit more irregular morphological boundaries, thereby increasing the difficulty of segmentation. Furthermore, obtaining large-scale, high-quality pixel-level annotations for follow-up NCCT generally involves substantial expert annotation effort, resulting in limited availability of labeled data for model training.

Semi-supervised learning offers a viable approach to addressing the aforementioned challenges by training jointly on a small number of labeled samples and a large number of unlabeled samples (19). Early consistency regularization methods, such as Temporal Ensembling (20) and Mean Teacher (21), have achieved significant success in image classification. When extended to semantic segmentation, they require applying the same data augmentation to both teacher and student models to avoid pixel-level spatial misalignment (22, 23). To prevent performance bottlenecks caused by strong coupling between teacher and student, Ke et al. proposed the Dual Student framework (24), enabling two structurally identical but differently initialized models to learn from each other by exchanging predictions on unlabeled samples. Subsequent approaches like Guided Collaborative Training (GCT) (25) introduced a defect detection branch to refine pseudo-labels, while Cross Pseudo Supervision (CPS) (26) directly used one model’s predictions as pseudo-labels for the other and applied CutMix for strong augmentation. More recent studies have explored alternative strategies for exploiting unlabeled data in semi-supervised medical image segmentation. BCP (27) employs bidirectional copy-paste between labeled and unlabeled samples within a Mean Teacher framework to reduce their empirical distribution gap. PICK (28) adopts a predict-and-mask strategy that masks pseudo-label-guided attentive regions in unlabeled images and reconstructs them through masked image modeling to improve the utilization of unlabeled data. Despite these advances, reliable exploitation of unlabeled data remains challenging in follow-up NCCT segmentation for ischemic stroke, particularly when manually labeled data are extremely limited. Our preliminary experiments with representative teacher–student and cross-pseudo-supervision methods reveal two major challenges in this task:

1. Severe overfitting under extremely limited annotation. Ischemic lesion segmentation is a foreground-background binary classification task. When the number of labeled samples is extremely limited (e.g., labeled samples only account for 1/16 of the entire training set), the model is highly prone to overfitting when repeatedly utilizing the limited labeled samples. Conventional affine transformation-based augmentation, including rotation and flipping, may provide insufficient sample diversity.
2. Training collapse caused by unreliable pseudo-labels. Pseudo-labeling approaches based on teacher models with exponential moving average (EMA) or dual-model mutual supervision may produce low-confidence and noisy pseudo-labels during the early training stage or when processing difficult low-contrast samples. Unreliable supervision signals cause the two models to mislead each other during mutual learning, leading to rapid performance degradation, which further exacerbates overfitting and performance fluctuations.

To address the aforementioned problems, this study proposes an ensemble-based semi-supervised segmentation (ESS) framework. Built upon the parallel dual-student model, the proposed framework introduces a strong data augmentation strategy (29, 30) to expand the diversity of labeled samples, and designs a parameter-free ensemble module that generates more stable and reliable pseudo-labels for unlabeled data by fusing the predictions of the two models, thereby effectively suppressing noise propagation. Unlike methods that directly perform cross-pseudo supervision between two student models, ESS combines their predictions before pseudo-label generation and uses the resulting unified ensemble pseudo-label to supervise both students.

The main contributions of this study are summarized as follows:

1. We design a parameter-free ensemble supervision mechanism that restructures the pseudo-label supervision pathway within the dual-student framework, reducing the direct propagation of erroneous predictions from an individual student.
2. To address unstable pseudo-label supervision and insufficient labeled-data diversity in follow-up NCCT segmentation under limited annotation, we develop the ESS framework by combining ensemble pseudo-label supervision with CutMix-based (31, 32) strong augmentation to improve supervisory reliability and training-sample diversity, respectively.
3. Based on real-world clinical data, we construct a large-scale follow-up NCCT dataset for ischemic stroke and systematically compare multiple semi-supervised segmentation methods. Experimental results demonstrate that ESS exhibits a pronounced performance advantage under extremely limited annotation.

## 2 The proposed approach

In this section, we define a single NCCT image as $X\in {\mathbb{R}}^{H\times W}$ , where H and W denote the height and width of the image, respectively. The objective of semi-supervised segmentation is to predict the label map $\hat{Y}\in {\left\{0,1\right\}}^{H\times W}$ for each pixel, where 0 represents the background and 1 represents the ischemic lesion. The training set D contains N labeled samples and M unlabeled samples, with N<<M , and can be represented as two subsets:


$$D=D^{\ell }\cup D^{u},$$

$D^{\ell }={\left\{(X_{i}^{l},Y_{i}^{l})\right\}}_{i=1}^{N},$ $D^{u}={\left\{X_{i}^{u}\right\}}_{i=N+1}^{N+M}.$

### 2.1 Overview of ESS

The overall workflow of the proposed Ensemble-based Semi-Supervised Segmentation (ESS) framework is illustrated in Figure 1. The framework adopts a dual-student parallel architecture. In each training batch, we randomly sample two labeled images $\left(X_{a}^{l},X_{b}^{l}\right)$ from the labeled dataset $D^{\ell }$ , and two unlabeled images $\left(X_{a}^{u},X_{b}^{u}\right)$ from the unlabeled dataset $D^{u}$ . We then randomly pair the two labeled images with each other and the two unlabeled images with each other, and apply CutMix augmentation to generate two mixed images, denoted as ${\tilde{X}}^{l}$ and ${\tilde{X}}^{u}$ . These mixed images are fed simultaneously into the two student models to obtain two sets of segmentation probability maps.

**Figure 1:**
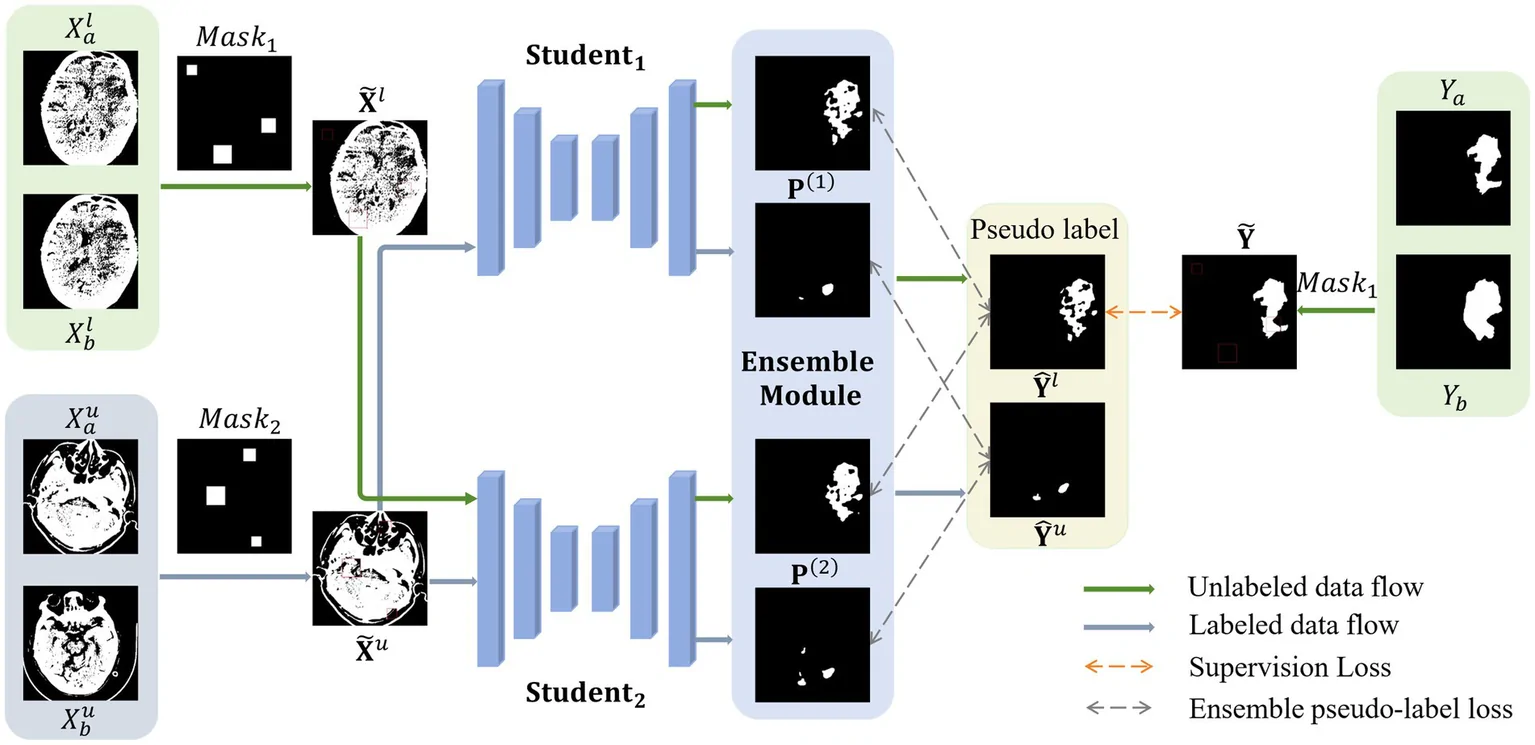
Ensemble-based semi-supervised segmentation (ESS) algorithm framework.

For both labeled and unlabeled samples, we employ a parameter-free ensemble module to fuse the outputs of the two models to generate more reliable ensemble pseudo-labels. Subsequently, each student model is trained under two types of supervision: (1) Supervised loss: on labeled samples, the weighted cross-entropy loss and Dice loss are calculated using ground truth labels; (2) Ensemble pseudo-label loss: for both labeled and unlabeled samples, the identical loss functions are computed using pseudo-labels generated by the ensemble module. Through this approach, unlabeled data are incorporated into model optimization through ensemble pseudo-label supervision, and the total loss function is defined as the weighted sum of all components.

In the inference stage, we average the lesion probability maps $P_{1}$ and $P_{2}$ generated by the two student models to obtain the final prediction probability map, formulated as:


$$P_{\text{final}}=\frac{P_{1}+P_{2}}{2}$$

and pixels with $P_{\text{final}}>0.5$ are classified as ischemic lesions.

### 2.2 Dual student structure

Let the two student models be defined as $f_{\theta_{1}}$ and $f_{\theta_{2}}$ which share the same DeepLabv3+ (33–35) network architecture but are initialized with different parameters, i.e., $\theta_{1}\neq \theta_{2}$ . The two student models do not share model parameters and are optimized independently during training. Unlike conventional teacher-student methods such as Mean Teacher, ESS does not adopt exponential moving average, and both models are directly optimized via gradient descent. This design avoids excessive coupling between the teacher model and the student model, thereby alleviating the performance bottleneck (22).

For an input image X , the two models output the segmentation probability maps respectively:


$$P^{\left(1\right)}=\text{Sigmoid}(f_{\theta_{1}}(X))$$

$$P^{\left(2\right)}=\text{Sigmoid}(f_{\theta_{2}}(X))$$

where $P^{\left(k\right)}\in {\left[0,1\right]}^{H\times W}$ , k∈{1,2} . The probability maps represent the predicted lesion probabilities at the corresponding spatial positions and serve as inputs to the ensemble pseudo-label generation module described in Section 2.4.

### 2.3 Data augmentation strategy

CutMix (31, 32) is a region-level data augmentation approach that mixes regions of two images to generate new samples, which can effectively alleviate overfitting. In the proposed framework, CutMix is only applied to samples of the same type: labeled images are mixed with other labeled images, while unlabeled images are mixed with other unlabeled images. The specific implementation is described below.

For each pair of images to be mixed, a binary mask $M\in {\left\{0,1\right\}}^{H\times W}$ is generated. The mask is constructed using K randomly located rectangular regions, with K=3 in this study. For the k−th rectangle, a scale parameter $r_{k}$ is uniformly sampled from [0.25,0.5] . Considering the K rectangular regions, the normalized width and height are both scaled by $1/\sqrt{K}$ , and the aspect ratio is fixed at 1:1. The spatial position of each rectangular region is randomly determined while ensuring that it remains within the image boundaries. Thus, the binary mask determines the spatial regions exchanged between the two images. Given two images $X_{a}$ and $X_{b}$ , the mixed image is defined as:


$$\tilde{X}=M⊙X_{a}+(1-M)⊙X_{b}$$

where ⊙ denotes element-wise multiplication, and 1 represents an all-ones matrix. If both mixed images are labeled samples, the corresponding ground-truth labels should also be mixed with the same mask:


$$\tilde{Y}=M⊙Y_{a}+(1-M)⊙Y_{b}$$

For unlabeled samples, the binary mask is used only to construct the mixed image. Since ground-truth labels are unavailable, pseudo-labels are neither predefined nor mixed during the CutMix operation. Instead, the mixed unlabeled image is first processed by the two student models, and its pseudo-label is subsequently generated from their predictions using the ensemble module described in Section 2.4.

Independent random masks are generated for the labeled and unlabeled data streams. Consequently, CutMix increases local sample diversity without introducing input–supervision inconsistency.

### 2.4 Ensemble pseudo-label generation module

The main methodological difference between ESS and direct cross-pseudo-supervision methods lies in how pseudo-labels are generated and transferred. In CPS, the pseudo-label derived from one student model is directly used to supervise the other student, and CPS with CutMix retains this cross-supervision mechanism while modifying the input samples through regional mixing. ESS instead introduces an explicit prediction-level ensemble before pseudo-label generation. The predictions of the two students for the same input are first fused, and a unified pseudo-label is subsequently generated and used to supervise both student models. Unlike an additional auxiliary network or learnable fusion branch, the proposed ensemble module contains no trainable parameters and operates only on the output probability maps.

For a spatial position (i,j) , let $p_{\mathrm{ij}}^{\left(1\right)},p_{\mathrm{ij}}^{\left(2\right)}\in [0,1]$ denote the lesion probabilities predicted by the first and second student models, respectively. Accordingly, $1-p_{\mathrm{ij}}^{\left(1\right)}$ and $1-p_{\mathrm{ij}}^{\left(2\right)}$ denote their corresponding background probabilities. Two ensemble strategies are considered: maximum-confidence and voting.

For the maximum-confidence strategy, the foreground and background confidence scores are defined as


$$S_{\mathrm{ij}}^{\mathrm{fg}}=\max(p_{\mathrm{ij}}^{\left(1\right)},p_{\mathrm{ij}}^{\left(2\right)})$$

$$S_{\mathrm{ij}}^{\mathrm{bg}}=\max(1-p_{\mathrm{ij}}^{\left(1\right)},1-p_{\mathrm{ij}}^{\left(2\right)})$$

The final binary ensemble pseudo-label is then determined by


$${\hat{Y}}_{\mathrm{ij}}=\{\begin{array}{cc} 1, & S_{\mathrm{ij}}^{\mathrm{fg}}>S_{\mathrm{ij}}^{\mathrm{bg}},\\ 0, & \text{otherwise}, \end{array}$$

where ${\hat{Y}}_{\mathrm{ij}}=1$ denotes the ischemic lesion and ${\hat{Y}}_{\mathrm{ij}}=0$ denotes the background. This strategy selects the class associated with the highest confidence among the lesion and background predictions of the two student models.

For the voting strategy, the lesion and background probabilities predicted by the two student models are aggregated as


$$V_{\mathrm{ij}}^{\mathrm{fg}}=p_{\mathrm{ij}}^{\left(1\right)}+p_{\mathrm{ij}}^{\left(2\right)}$$

$$V_{\mathrm{ij}}^{\mathrm{bg}}=(1-p_{\mathrm{ij}}^{\left(1\right)})+(1-p_{\mathrm{ij}}^{\left(2\right)})$$

The voting-based pseudo-label ${\hat{Y}}_{\mathrm{ij}}^{\text{vote}}$ ​ is defined as


$${\hat{Y}}_{\mathrm{ij}}^{\text{vote}}=\{\begin{array}{cc} 1, & V_{\mathrm{ij}}^{\mathrm{fg}}>V_{\mathrm{ij}}^{\mathrm{bg}},\\ 0, & \text{otherwise}, \end{array}$$

Here, ${\hat{Y}}_{\mathrm{ij}}^{\text{vote}}$ ​ denotes the binary pseudo-label generated by the voting strategy at spatial position (i,j) . Unlike the maximum-confidence strategy, the voting strategy aggregates the predictions of both student models for the same class and determines the final class according to the aggregated probabilities.

The experimental results presented in Section 3 demonstrate that the maximum-confidence strategy yields higher Dice coefficients than the voting strategy at all annotation ratios. Therefore, maximum-confidence is adopted as the default strategy for ESS.

The ensemble module is applied to both labeled and unlabeled samples. For labeled samples, the ensemble module generates pseudo-labels used in the ensemble pseudo-label loss, serving a regularization role; for unlabeled samples, the pseudo-labels generated by the ensemble module constitute their sole supervisory signal. For each input, the same unified pseudo-label generated from the predictions of both student models is used to supervise the two students. Pseudo-labels are dynamically computed within each training batch and are not cached for subsequent batches.

### 2.5 Loss function

The total loss function of ESS consists of two components: the supervised loss and the ensemble pseudo-label loss. For a labeled sample, let $P^{\left(k\right)}$ denote the probability map predicted by the k -th student and $\tilde{Y}$ denote the corresponding ground-truth label after CutMix. The supervised loss is defined as the sum of the Dice loss and the weighted cross-entropy loss. For each student model k ,


$$L_{\sup}^{\left(k\right)}=(L_{\text{dice}}(P^{\left(k\right)},\tilde{Y})+L_{\mathrm{wce}}(P^{\left(k\right)},\tilde{Y}))$$

The supervised losses of the two students are summed as


$${ℒ}_{\sup}=\sum_{k=1}^{2}{ℒ}_{\sup}^{\left(k\right)}$$

The ensemble pseudo-label loss adopts the standard cross-entropy loss. For any given image, let $\hat{Y}$ denote the pseudo-label generated by the ensemble module, and P denote the probability map predicted by the model. The cross-entropy loss is defined as follows:


$$L_{\mathrm{ce}}(P,\hat{Y})=-\frac{1}{\mathrm{HW}}\sum_{i,j}[{\hat{Y}}_{\mathrm{ij}}\log P_{\mathrm{ij}}+(1-{\hat{Y}}_{\mathrm{ij}})\log(1-P_{\mathrm{ij}})]$$

For unlabeled samples, the pseudo-label loss of each student model is defined as $L_{\mathrm{ce}}(P^{\left(k\right)},{\hat{Y}}^{u})$ , where ${\hat{Y}}^{u}$ denotes the ensemble pseudo-label generated by the predictions of the two models for the unlabeled samples. The total pseudo-label loss on all unlabeled samples is defined as:


$$L_{\mathrm{pl}}^{u}=\sum_{k=1}^{2}L_{\mathrm{ce}}(P^{\left(k\right)},{\hat{Y}}^{u})$$

For labeled samples, the pseudo-label loss ${ℒ}_{\mathrm{pl}}^{l}$ is computed in the same manner as that for unlabeled samples, and the total pseudo-label loss is the sum of the two terms.


$${ℒ}_{\mathrm{pl}}={ℒ}_{\mathrm{pl}}^{l}+{ℒ}_{\mathrm{pl}}^{u}$$

The total loss function of ESS is the sum of the supervised loss and the weighted pseudo-label loss,


$${ℒ}_{\text{total}}={ℒ}_{\sup}+\lambda {ℒ}_{\mathrm{pl}}$$

where λ controls the contribution of the ensemble pseudo-label loss and is set to 2 in our experiments.

## 3 Experiments

### 3.1 Dataset and evaluation metrics

In this study, a total of 1,004 follow-up NCCT cases were collected from 721 patients with ischemic stroke across 18 comprehensive stroke centers nationwide. Lesion annotations were independently reviewed by two radiologists who were blinded to the patients’ clinical information. Any disagreement regarding lesion extent or boundaries was resolved by consensus after joint review, and the consensus result was used as the final annotation. After data preprocessing and quality control, a total of 9,020 image slices were retained. The training/test partition was performed at the case level with an approximate ratio of 4:1 while strictly maintaining patient-level independence. All cases and corresponding slices from the same patient were assigned exclusively to either the training set or the test set, ensuring that no patient overlap existed between the two subsets. The resulting training and test sets comprised 7,216 and 1,804 image slices, respectively. The training set was further divided into labeled and unlabeled subsets under four experimental configurations, with the proportions of labeled samples set to 1/2, 1/4, 1/8, and 1/16. These labeled/unlabeled subsets were constructed at the slice level within the training set. The corresponding numbers of labeled and unlabeled samples are summarized in Table 1.

**Table 1:** Number of labeled and unlabeled samples under four labeled-data ratios.

The ratio of labeled samples	The number of labeled samples	The number of unlabeled samples
1/2	3,608	3,608
1/4	1,804	5,412
1/8	902	6,314
1/16	451	6,765

During training, data augmentation via affine transformation is applied with a probability of 0.8, including horizontal flipping, vertical flipping and random rotation. The input image size is fixed at 400 × 400 pixels.

Segmentation performance is evaluated using the Dice coefficient and Intersection over Union (IoU). The Dice coefficient is defined as:


$$\text{Dice}=\frac{2\times \mathrm{TP}}{2\times \mathrm{TP}+\mathrm{FP}+\mathrm{FN}}$$

IoU is defined as:


$$\mathrm{IoU}=\frac{\mathrm{TP}}{\mathrm{TP}+\mathrm{FP}+\mathrm{FN}}$$

Both indicators quantify the overlap degree between the predicted segmentation and the ground-truth labels, where higher values indicate better consistency. In this study, the positive class corresponds to ischemic lesions, whereas the negative class corresponds to background regions.

### 3.2 Implementation details

All experiments were implemented in PyTorch 1.10.0 with Python 3.8. The comparative methods include Mean Teacher (MT) (21), GCT (25), CPS (26), CPS with CutMix, BCP (27), and PICK (28).

For MT, GCT, CPS, CPS with CutMix, and the proposed ESS, DeepLabv3 + with an ImageNet-pretrained ResNet-50 backbone was adopted as the segmentation network, with an output stride of 16. These methods were implemented using a unified segmentation architecture to reduce the influence of backbone differences and facilitate comparison of their semi-supervised learning strategies.

For BCP and PICK, the method-specific network architectures, training strategies, and hyperparameter configurations of their original implementations were retained. Only dataset-related components were adapted to the follow-up NCCT dataset, including data loading and the construction of labeled and unlabeled subsets. This setting avoids substantial modification of their original method-specific designs.

ESS was optimized using the Adam optimizer with an initial learning rate of 1 × 10^−4^ and a weight decay of 1 × 10^−4^, and β1 = 0.9 and β2 = 0.999. The batch sizes of the labeled and unlabeled data were both set to 4. The weighting coefficient λ for the ensemble pseudo-label loss was set to 2. Training was conducted for 20 epochs.

Across all experiments, the test set was evaluated after each epoch without being involved in model training or parameter optimization, and the best Dice performance observed within the predefined training schedule was reported. Experiments were conducted on two NVIDIA GeForce RTX 3080 GPUs, and the Dice coefficient was adopted as the primary evaluation metric.

### 3.3 Experimental results and analysis

This section evaluates the proposed ESS against several semi-supervised segmentation methods, including Mean Teacher, GCT, CPS, CPS with CutMix, BCP, and PICK, under four annotation ratios.

When the annotation ratio is 1/16, the segmentation performance of MT and GCT is even lower than that of the baseline model trained only with labeled data. CPS achieves performance improvement via cross pseudo supervision. After introducing CutMix, the Dice coefficient of CPS increases by 1.90, 1.53, 0.05, and 0.17 percentage points under the four experimental settings, respectively. These results indicate that CutMix provides additional performance gains, with larger improvements observed under lower annotation ratios and only marginal gains under higher annotation ratios.

As shown in Figure 2, the severe class imbalance between labeled and unlabeled samples leads to a continuous decline in the IoU and Dice coefficient of unlabeled samples. This indicates that directly using the output of one model as the pseudo-label for another model during Cross Pseudo Supervision (CPS) training introduces noisy supervision, which results in unstable information exchange. As the training progresses, the quality of pseudo-labels deteriorates continuously, making it difficult for the model to learn effective features from unlabeled data.

**Figure 2:**
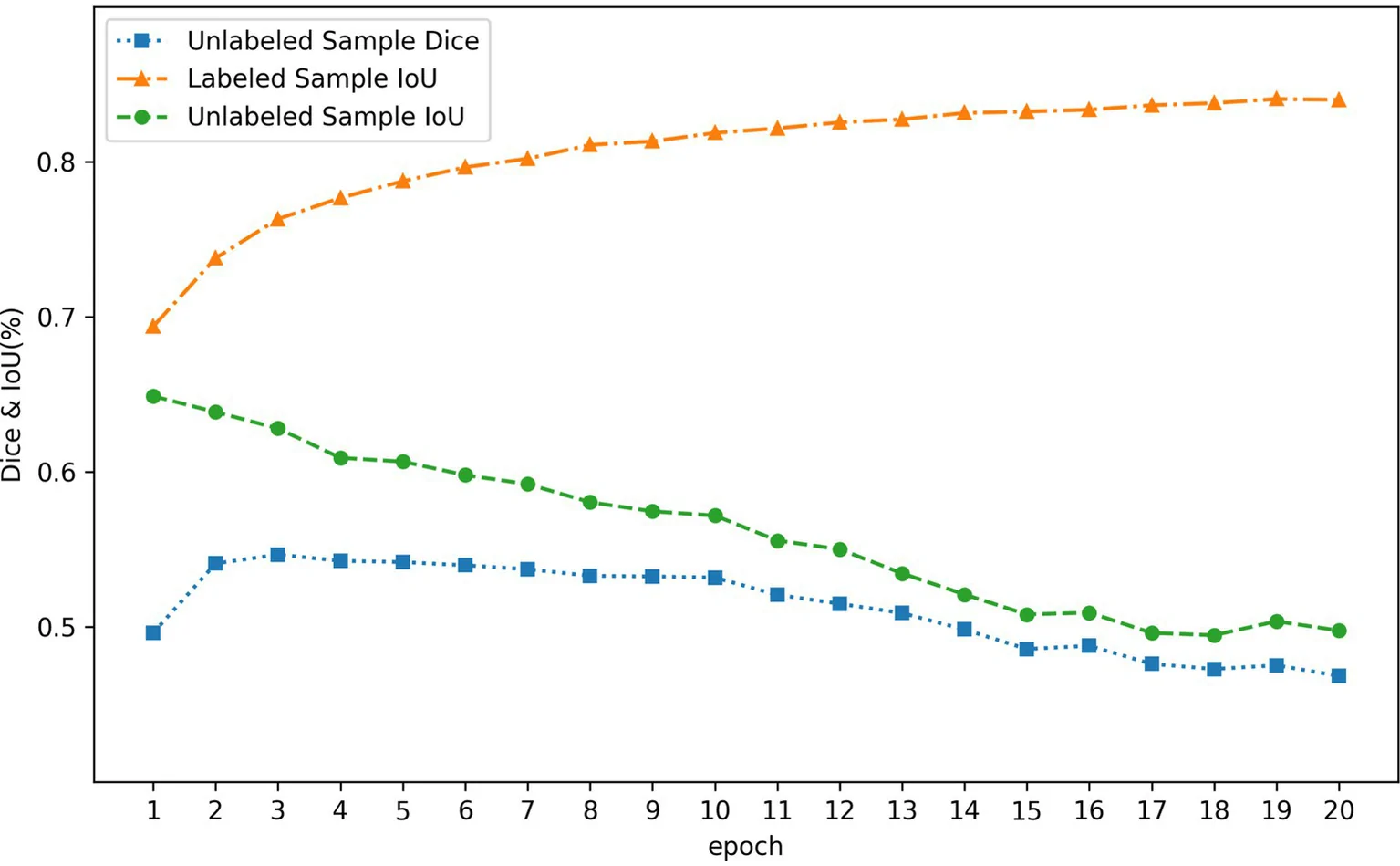
Dice of unlabeled samples and IoU of labeled and unlabeled samples during CPS training under the 1/8 annotation setting.

Table 2 presents the Dice coefficients of the proposed ESS, the Baseline method, and existing semi-supervised methods under four different annotation ratios. Specifically, the Baseline refers to the DeepLabv3 + model trained exclusively on labeled data.

**Table 2:** Comparison of segmentation results between ESS and other semi-supervised algorithms.

Method	1/16	1/8	1/4	1/2
Baseline	47.48	51.65	55.77	59.03
Mean teacher	37.28	50.03	54.87	57.34
GCT	36.21	45.39	50.35	56.78
CPS w/o CutMix	41.86	50.06	56.92	60.53
CPS w/CutMix	43.76	51.59	56.97	60.70
BCP	46.00	48.66	51.06	52.13
PICK	44.94	49.40	50.80	51.44
ESS	49.85	53.47	58.52	61.21

As shown in Table 2, ESS achieves the highest Dice coefficient across all four experimental setups. Compared with the baseline model, ESS improves the Dice coefficient by 2.37, 1.82, 2.75, and 2.18 percentage points under the four experiments, respectively. In comparison with the conventional semi-supervised method CPS with CutMix, ESS improves Dice coefficient by 6.09, 1.88, 1.55, and 0.51 percentage points, respectively. ESS also achieves higher Dice coefficients than the recent semi-supervised methods BCP and PICK under all four annotation ratios. Overall, the performance advantage of ESS is most pronounced under the 1/16 setting and becomes more modest as the proportion of labeled data increases.

In Section 2.4 we introduce two ensemble algorithms: the maximum-confidence method and the voting method. Table 3 presents the segmentation results of ESS under the maximum-confidence ensemble strategy and the voting ensemble strategy. CutMix is not used in this experiment. The baseline model corresponds to DeepLabv3 + trained only with labeled data.

**Table 3:** Comparison of segmentation results under different ensemble strategies.

Method	1/16	1/8	1/4	1/2
Baseline model	47.48	51.65	55.77	59.03
ESS and voting	49.03	52.03	55.68	59.96
ESS and maximum-confidence	49.52	53.17	56.38	60.62

The results indicate that the maximum-confidence strategy yields higher Dice coefficients than the voting strategy at all annotation ratios, with improvements of 0.49, 1.14, 0.70, and 0.66 percentage points, respectively. Although the improvement is relatively modest in some settings, the maximum-confidence strategy consistently achieves better performance. Based on the aforementioned comparisons, ESS selects the maximum-confidence strategy as the default ensemble strategy.

Figure 3 compares the segmentation performance of various methods on lesions at different scales. For large-volume lesions with relatively clear boundaries shown in the first three rows of the figure, all methods can achieve satisfactory segmentation results. However, for small lesions presented in the last three rows, the performance of different methods varies considerably. Compared with other methods, the ESS proposed in this paper can still maintain better segmentation performance on these more challenging small lesions.

**Figure 3:**
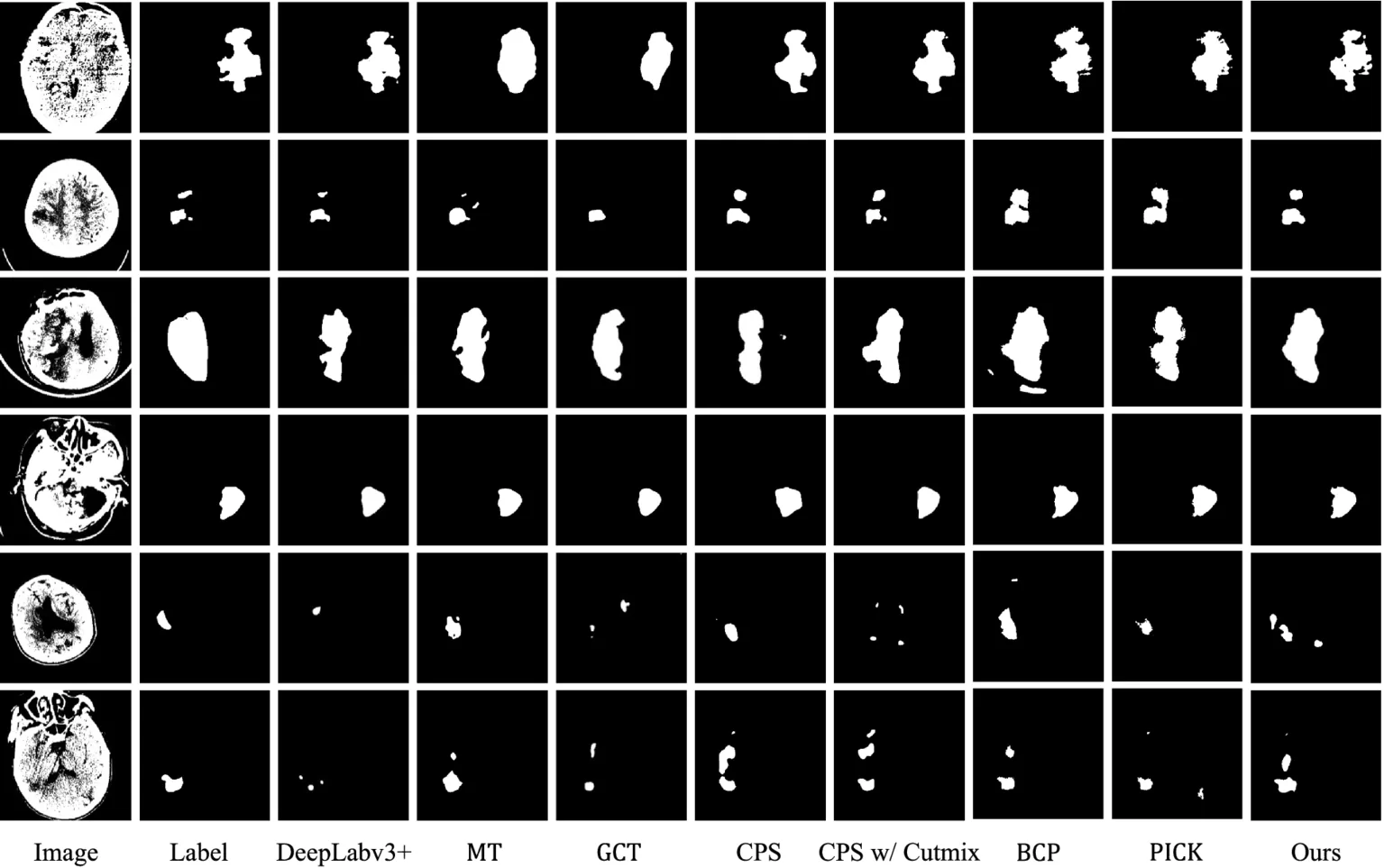
Visualization comparison of segmentation results of various models on typical follow-up NCCT images under 1/8 annotation ratio.

As shown in Figure 4, compared with CPS, the IoU and Dice curves for unlabeled samples under ESS are more stable and attain higher values in the later training stage, indicating that the cross-model information exchange is more reliable, and the performance degradation on unlabeled data is effectively alleviated.

**Figure 4:**
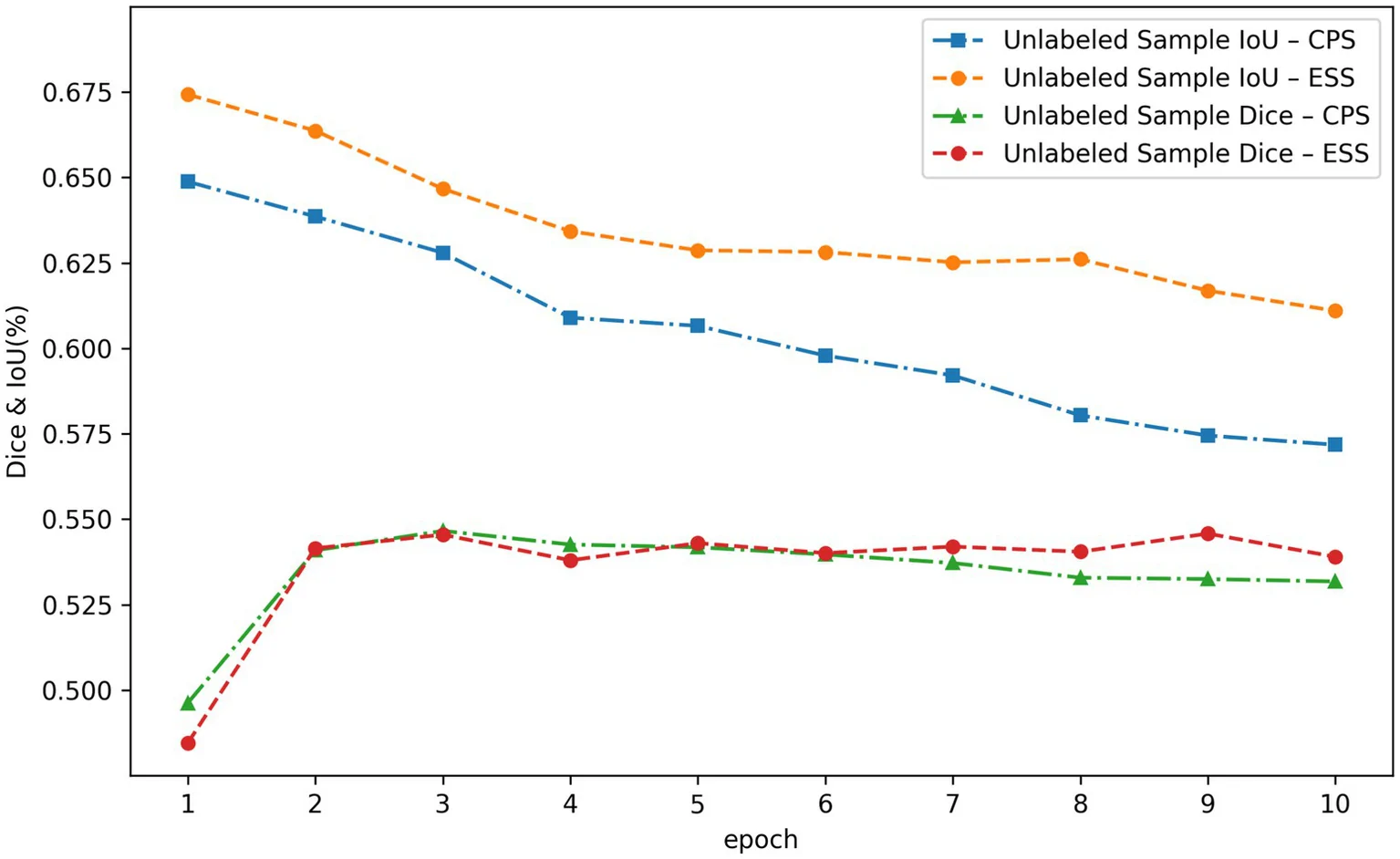
Dice and IoU curves of unlabeled samples during ESS and CPS training.

Table 4 presents the results of the ablation study conducted on the ESS method. The CPS model without CutMix is used as the baseline. The ensemble module denotes the proposed maximum-confidence ensemble pseudo-label generation mechanism, whereas CutMix indicates that the strong data augmentation method is applied to both labeled and unlabeled samples.

**Table 4:** Ablation study results of the ESS algorithm.

Method	Ensemble module	CutMix	1/16	1/8	1/4	1/2
Baseline			41.86	50.06	56.92	60.53
ESS w/o CutMix	√		49.52	53.17	56.38	60.62
ESS	√	√	49.85	53.47	58.52	61.21

As shown in Table 4, introducing the ensemble module improves segmentation performance under most annotation settings, with more pronounced gains when labeled data are limited. Further incorporating CutMix consistently improves the results across all four annotation ratios.

## 4 Discussion

Deep learning-based segmentation models typically require substantial pixel-level annotations, which are costly to obtain in medical imaging and motivate the use of semi-supervised learning (19). Existing semi-supervised segmentation approaches include Teacher–Student consistency frameworks (36) and dual-student cross-learning methods such as Dual Student (24, 36–38), GCT (25), and CPS (26). Although these methods exploit unlabeled data in different ways, the reliability of pseudo-label supervision remains a key challenge, particularly when labeled data are scarce. Recent supervised studies have also advanced ischemic stroke lesion segmentation through task-specific network architectures for NCCT and MRI (16–18). In contrast to these studies focusing on network architecture design, the present work focuses on improving follow-up NCCT segmentation under limited annotation by jointly exploiting labeled and unlabeled data.

In the follow-up non-contrast computed tomography (NCCT) image segmentation task for ischemic stroke, representative semi-supervised methods still face two practical challenges under limited annotation. First, the model is prone to overfitting when only a small amount of labeled data is available. Second, pseudo-labels generated via direct cross-learning are often unreliable, leading to unstable supervision for unlabeled samples. To address these issues, ESS combines strong data augmentation with ensemble pseudo-label supervision to improve training-sample diversity and pseudo-label reliability, respectively.

Experimental results show that the performance gain of ESS varies with the annotation ratio. Compared with CPS with CutMix, ESS improves the Dice coefficient by 6.09 percentage points under the 1/16 setting, whereas the improvement decreases to 0.51 percentage points under the 1/2 setting. This trend indicates that the additional benefit of ESS is more pronounced when labeled data are scarce and becomes more limited as supervised information increases. Nevertheless, ESS achieves the highest Dice coefficient among the compared methods under all four annotation ratios. BCP (27) and PICK (28) yield lower Dice scores than the supervised baseline on the present dataset. Because their method-specific configurations were retained, these results may partly reflect differences between their original task settings and the present follow-up NCCT task. The ablation results further show that the ensemble module provides larger gains under lower annotation ratios, while the incorporation of CutMix yields additional improvements across all evaluated settings.

Nevertheless, this study still has certain limitations. Although the experiments under four annotation ratios support the effectiveness of ESS in the investigated follow-up NCCT setting, the evaluation is limited to a single dataset without independent external validation; therefore, its generalizability across different datasets remains to be further established. All experiments were conducted with a fixed random seed, and the standard deviation of multiple repeated experiments is not reported. In addition, ESS still produces segmentation errors under extremely low labeling ratios. For small lesions in particular, pseudo-label noise may affect segmentation boundary accuracy. Future work will evaluate ESS on additional independent datasets, conduct repeated experiments to evaluate statistical significance, and further improve the segmentation performance for small lesions.

## 5 Conclusion

In this study, we analyze the limitations of existing semi-supervised segmentation methods when applied to the segmentation of follow-up non-contrast computed tomography (NCCT) images of ischemic stroke, with particular emphasis on the overfitting issue under limited annotation and the instability of pseudo-label generation in cross-learning frameworks. Based on these observations, we propose ESS, an ensemble-based semi-supervised segmentation method that integrates strong data augmentation and an ensemble pseudo-label strategy.

By mitigating overfitting and improving the reliability of pseudo-labels, ESS improves segmentation performance under different annotation ratios. Experimental results show that ESS achieves the highest Dice coefficient among the compared methods under all four annotation ratios, with more pronounced gains when labeled data are scarce and more modest gains at higher annotation ratios. Ablation experiments further support the contribution of the ensemble module and the additional performance improvement obtained by incorporating CutMix. These results demonstrate the effectiveness of ESS for semi-supervised segmentation of follow-up NCCT under limited annotation conditions, while its generalizability should be further evaluated on independent datasets.

## Data Availability

The data analyzed in this study is subject to the following licenses/restrictions: The clinical NCCT dataset used in this study is not publicly available due to patient privacy and institutional data security regulations. However, de-identified data may be made available from the corresponding author upon reasonable request, subject to ethical approval and data transfer agreements. Requests to access these datasets should be directed to Long Wang, lwang@ustb.edu.cn.
